# 20. Neurovascular BehÇet’s syndrome: lessons in management from a centre of excellence

**DOI:** 10.1093/rap/rky033.012

**Published:** 2018-09-20

**Authors:** Lauren A Dolan, Priyanka Chandratre

**Affiliations:** Rheumatology, Sandwell and West Birmingham NHS Trust, Birmingham, UNITED KINGDOM

**Introduction:** Within the UK, Behçet’s syndrome has an estimated prevalence of 1-2 cases per 100, 000 population. Neurological manifestations were estimated to affect 13% of patients following systematic review upon writing the 2018 EULAR guidelines, whereas vascular involvement was estimated to affect 22% and typically occurs within the first five years of fulfilling International Study Group criteria with a median duration of time to first vascular event of 1.4 years. Publication of EULAR guidelines has led to much debate surrounding recommended best practice when managing such manifestations; particularly in the case of recurrent vascular events which can affect up to 35.4% of patients with vascular Behçet’s. Here we present a case of recurrent venous sinus thrombosis on a background of recurrent venothromboembolic episodes secondary to Behçet’s disease.

**Case description:** A thirty-seven year old female with refractory vascular Behçet’s first presented to our tertiary centre in October 2013. Initial presentation with recurrent orogenital ulceration and erythema nodosum at age thirteen had been managed with good response with ciclosporin and colchicine but in 2006 the patient developed recurrent deep venous thromboembolisms (DVTs) and pulmonary embolisms requiring anticoagulation with warfarin and an inferior vena cava filter. In 2012, the patient underwent gastric banding, incidentally following which ciclosporin was ineffective at controlling mucocutaneous disease and she was referred to our tertiary centre for further opinion. Upon review, the patient also reported migraines and arthralgia previously requiring intraarticular injection. She was married with three children having suffered no previous pregnancy complications and smoked ten cigarettes per day. Azathioprine was commenced but persistent nausea led to discontinuation. Following reluctance to try an alternative disease modifying therapy, colchicine was successfully recommenced with marked improvement in orogenital ulceration. In March 2015, the patient presented with an eleven-day history of headache but no visual changes. Cerebral sinus thrombosis was strongly suspected and although CT head revealed a small filling defect, this was felt to represent arachnoid granulations after MRI brain excluded a venous sinus thrombosis. Throughout the investigation period, mycophenolate mofetil (MMF) alongside prednisolone 15 mg OD had been commenced for suspected active disease; she continued this alongside colchicine and anticoagulation therapy (target INR 2-3). Within a month, the patient represented with sudden painless loss of vision in the right eye. Ophthalmic assessment diagnosed right retinal vein embolus with mild inferior retinal vasculitis representing active, sight threatening, vasculitic disease. Treatment was escalated to induction therapy with cyclophosphamide (alongside pulsed intravenous methylprednisolone), followed by maintenance with MMF 2g/day and prednisolone 10 mg/day. Subsequently, tertiary centre neuroradiology review of initial CT head confirmed a very small thrombus in the right transverse sinus; thus, identifying the cause of headache and demonstrating the importance of obtaining expert review of imaging, especially in view of an evolving clinical picture. In June 2016, whilst maintained on MMF and prednisolone 10 mg OD, the patient suffered a further venous sinus thrombosis, presenting to the acute medical team at her local district general hospital. It was felt secondary to subtherapeutic anticoagulation (international normalised ratio (INR) 1.4) despite home monitoring and warfarin dosing. Target range INR was consequently increased to four and the headache slowly resolved. Upon Behçet’s clinic review two months later, there had been no other disease manifestations and orogenital ulceration was noted to be improving; prednisolone was slowly weaned to 5 mg a day. Unfortunately, over the subsequent year symptoms of orogenital ulceration and arthralgia increased and in March 2018 the patient presented acutely with left sided headache, left temporal field hemianopia and left sided hearing loss. MRI confirmed a non-occlusive thrombus in the left sigmoid sinus which had occurred despite an INR >6.0. Subsequently, immunosuppression was escalated to pulsed intravenous methylprednisolone followed by high dose oral prednisolone (30 mg OD) and infliximab which the patient is tolerating well.

**Discussion:** Cerebral venous sinus thrombosis is classified as a neurological manifestation of Behçet’s disease by the latest EULAR 2018 guidelines, initial presentation should be managed with high dose intravenous glucocorticoids but there is little data to support addition of immunosuppression as relapses in the condition are not thought to be frequent. Our case is unusual in that multiple acute cerebral venous sinus thrombosis have occurred on a background of previous venothromboembolic disease and anticoagulation. The use of anticoagulation in cerebral venous sinus thrombosis is supported for a short duration, particularly if patients pose additional prothrombotic risk factors such as obesity or current smoking; as in our case. When faced with the clinical conundrum of recurrent cerebral venous thrombosis, the multidisciplinary team chose to extrapolate current guidelines surrounding refractory venous thrombosis whereby recurrent events clearly require escalation in immunosuppression to include cyclophosphamide and monoclonal anti-TNF. Recommendations for anticoagulation in refractory venous thrombosis are not clearly defined; anticoagulation alongside immunosuppression has not been proven to reduce the risk of further venothromboembolic events in Behçet’s disease when compared to immunosuppression alone, but may reduce the risk of post-thrombotic syndrome which can result in devastating leg ulceration refractory to treatment. Overall, there is lack of evidence to advise for or against the use of anticoagulation long term, but it is deemed likely appropriate in cases of low haemorrhagic risk where pulmonary artery aneurysms (often observed in patients with a background of DVT’s) have been excluded.

**Key Learning Points:** This case highlights three key learning points. Firstly, the importance of specialist radiology review should the clinical picture and imaging not align. Secondly, the importance of liaising with tertiary centres during an acute admission, as this may have led to escalation in treatment regimen following venous sinus thrombosis in 2016. The final learning point surrounds the careful balancing act required between immunosuppression and anticoagulant therapy required when treating both neurological and vascular manifestations of Behçet’s. Not only is it vital to ensure low bleeding risk and absence of pulmonary aneurysms prior to commencing anticoagulant therapy but this should be re-evaluated periodically and should never act as substitute for immunosuppressive treatment.

**Disclosure: L.A. Dolan:** None. **P. Chandratre:** None.

